# An Unusual Cause of Right Heart Dysfunction and High Output Heart Failure in a Young Woman

**DOI:** 10.3390/jcdd9120418

**Published:** 2022-11-26

**Authors:** Nicolás Ariza Ordoñez, Antonia Pino Marín, Juan Sebastián Bonilla Crespo, Alberto Navarro Navajas, Gabriel Antonio Oliver, Hector M. Medina, Julián F. Forero

**Affiliations:** 1 Department of Internal Medicine, Fundación Cardioinfantil—La Cardio, Bogotá 110131, Colombia; 2 School of Medicine and Health Sciences, Universidad del Rosario, Bogotá 110131, Colombia; 3 Department of Cardiology, Fundación Cardioinfantil—La Cardio, Bogotá 110131, Colombia; 4 Division of Cardiovascular Imaging, Fundación Cardioinfantil—La Cardio, Bogotá 110131, Colombia; 5 Department of Radiology and Diagnostic Imaging, Fundación Cardioinfantil—La Cardio, Bogotá 110131, Colombia

**Keywords:** hereditary hemorrhagic telangiectasia (HHT), high output heart failure, cor triatriatum

## Abstract

A 35-year-old female presented to our emergency department with clinical signs of acute heart failure. Clinical workup identified severe right heart (RH) dilation and dysfunction with a crossing membrane structure in the right atrium. Right heart catheterization confirmed high output heart failure (HOHF), pulmonary hypertension (PH), and left-to-right blood shunting followed by the documentation of multiple liver and pulmonary arteriovenous malformations (AVMs). Hereditary Hemorrhagic Telangiectasia (HHT) diagnosis was made according to clinical criteria and was established as the cause of HOHF because of chronic volume overload from systemic to pulmonary shunts. With this illustrative case, we aim to discuss the broad spectrum of clinical manifestations of HHT and the unusual phenotype of HOHF secondary to HHT. This case also highlights the broad diagnosis of atrial echocardiographic abnormalities and cardiac structural distortion secondary to high output that can be misleading at imaging evaluation.

## 1. Introduction

HHT, also known as Osler–Weber–Rendu syndrome, is a rare autosomal dominant disorder characterized by the formation of multiple AVMs at different sites. Its estimated prevalence is reported to be 1 in 5000–10,000 [1]. Epistaxis and mucocutaneous AVMs are the most prevalent manifestations, however, larger AVMs in the liver, lungs, brain, and gastrointestinal tract can complicate the disease course [2]. The pathogenesis of HHT is related to mutations in genes encoding for transforming growth factor-β (TGF-β) receptors in endothelial cells. Up to 90% of cases are caused by mutations in endoglin (ENG; HHT type 1) or activin A receptor-like type 1 (ACVRL1 which encodes ALK1; HHT type 2), both components of the Bone Morphogenetic Protein 9 and 10 (BMP9/BMP10) signaling pathway [2].

Pathologic mutations result in decreased expression of ENG and ACVRL1, altering normal endothelial response to TGF-β/BMP signaling pathway; this leads to disruption of vascular integrity and smooth muscle differentiation resulting in abnormal cytoskeleton, fragile vessels, and AVM formation. Chronic inflammation and hypoxia worsen AVM apparition via vascular endothelial growth factor (VEGF) [3,4].

HHT has been associated with an increase in cerebrovascular and cardiovascular disease burden [5]. Cardiovascular complications of HHT include arterial hypertension, cardiac arrhythmias, and PH. HHT-related PH presents two main hemodynamic profiles: pulmonary arterial hypertension and HOHF, the latter mainly secondary to liver AVMs (abnormal communications between hepatic arteries, portal veins, and hepatic or systemic veins), which conditionate arteriovenous blood shunting. Liver AVMs can be identified in up to 40–75% of patients with HHT [6,7,8], leading to clinical manifestations in only 5–8% of patients with HHT [6,9,10,11].

We present the case of a young female patient, with a non-clear history of cor triatriatum (CTD) with congestive symptoms and a history of recurrent nasal bleeding. Pulmonary hypertension and HOHF with severe right atrial (RA) dilation and eustachian valve elongation mimicking CTD were documented. HOHF was attributed to chronic volume overload from arteriovenous shunt secondary to large hepatic AVMs complicating HHT diagnosis.

HOHF is a rare complication of HHT, most frequently caused by obesity, liver disease and arteriovenous shunts, especially AV fistulae for renal replacement therapy [12]. The pathogenesis of cardiac dysfunction in this scenario is associated with the shunting of oxygenated blood directly from the hepatic artery to the hepatic vein, decreasing effective perfusion. In consequence, there is an increased oxygen demand and decreased systemic vascular resistance which stimulate and enhance both the sympathetic nervous system and the renin-angiotensin-aldosterone system, causing greater cardiac output. The increase in venous return over time enhances RA pressure, pulmonary artery pressure and left ventricular (LV) end-diastolic volume, causing LV dilation and eventually cardiac failure [13].

## 2. Case Presentation

A 35-year-old Hispanic female presented with a 2-month history of exertional dyspnea, lower limb edema, orthopnea, palpitations, abdominal distention, and right upper quadrant abdominal pain. Vital signs on admission were normal and physical examination revealed clinical signs consistent with systemic congestion (ascites, edema, and crackles on auscultation) and several telangiectasias in the nasal and oral mucosa. The patient had a history of recurrent epistaxis, and a non-clear CTD diagnosis made 6 months ago; she also referred multiple hospitalizations due to ascites requiring paracentesis, which suggested a chronic hepatopathy of unknown etiology as its underlying disease. She underwent treatment with oral furosemide, spironolactone, and lactulose, with poor clinical response since her previous hospitalization.

**Figure 1:**
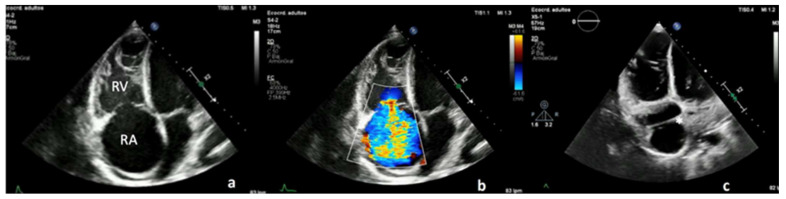
Transthoracic echocardiogram with severe right atrial (RA) and ventricular enlargement (**a**), severe tricuspid regurgitation (**b**) with an anomalous mobile membranous band-like structure crossing the RA (with asterisk in (**c**)). RV: Right ventricle RA: right atria.

Initial blood tests showed mild normocytic anemia and hyperbilirubinemia. Brain natriuretic peptide was elevated (566 pg/mL). Chest X-ray showed bilateral mild pleural effusions and signs of right cardiac chamber enlargement. Transthoracic echocardiogram revealed normal LV function, severe right ventricle (RV) dilation and dysfunction, and severe RA enlargement with an anomalous mobile membranous band-like structure crossing the RA with no significant trans-atrial gradient (Figure 1).

**Figure 2:**
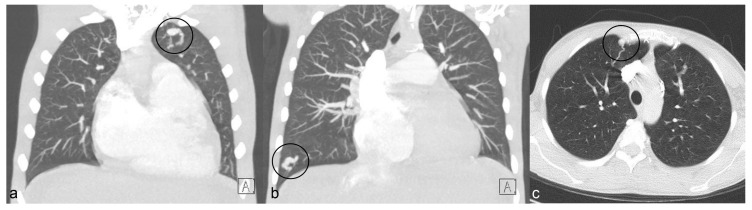
Contrast-enhanced chest CT. Coronal MIP (**a**,**b**) and axial (**c**) images show peripheral AVMs, seen as non-calcified nodules with a feeding arterial vessel (within black circle in **b**). MIP: Maximum Intensity Projection.

**Figure 3:**
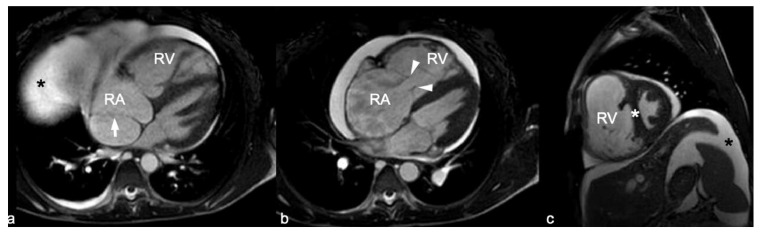
SSFP Cardiac MR images in the 4 chamber (**a**,**b**) and short axis (**c**) planes. RV and RA are severely dilated and diastolic septal shifting (white asterisk on **c**) as a sign of volume overload of the RV are seen. The eustachian valve is seen in the RA (White arrow on **a**) with no other membranous structures seen on more cephalic images. Severe tricuspid regurgitation with significant coaptation defect (between white arrowheads in (**b**)) was found, together with abdominal free fluid (Black asterisk on (**a**) and (**c**)), pericardial and pleural effusions. SSFP: Steady State Free Precession.

Chest computed tomography (CT) showed multiple homogeneous nodules in the bilateral upper and lower pulmonary lobes suggesting the presence of AVMs with no other parenchymal lung disease findings (Figure 2). Cardiac magnetic resonance (CMR) was performed to clarify the nature of the right chambers’ pathological changes, demonstrating severe tricuspid regurgitation (TR), marked dilation of both the RA and RV dilation, and severe RV dysfunction. The membranous structure previously described in the RA was considered to be a Chiari network or a prominent Eustachian valve (Figure 3) associated with severe RA enlargement rather than a subdivision into two separate chambers.

RH catheterization revealed PH with a mean pulmonary artery pressure of 29 mmHg (systolic pressure: 46 mmHg, diastolic pressure: 10 mmHg, measured by thermodilution) and normal pulmonary vascular resistance with mildly elevated pulmonary artery wedge pressure (16 mmHg). An elevated right cardiac index (5.55 L/min/m^2^), suggestive of HOHF, and a systemic to pulmonary shunt (Qp/Qs index: 2.4) were also identified.

**Figure 4:**
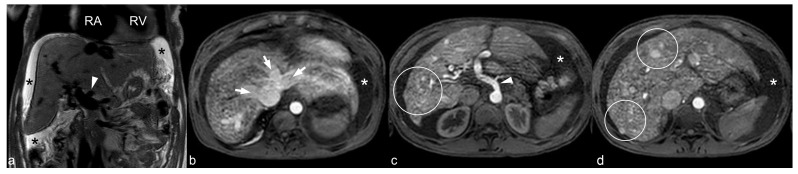
Abdominal MR. Coronal SSh image (**a**) and axial images on the arterial phase (**b**–**d**). Hepatomegaly and free abdominal fluid are seen (Black asterisk on (**a**) and white asterisk on (**b**–**d**). The hepatic veins as well as the inferior vena cava are dilated and show early enhancement (white arrows) due to extensive arteriovenous shunts and heterogeneous enhancement (within white circles). Due to these shunts the hepatic artery is dilated (white arrowheads) and multiple arteries are seen on the liver hilum. SSh: Single Shot.

An abdominal MRI was requested to further evaluate potential sources of an extracardiac blood shunting as well as liver morphology. It demonstrated multiple hepatic vascular lesions compatible with AVMs with no signs of liver parenchymal disease (Figure 4). Other in-patient studies included a pelvic CT which displayed bilateral parauterine AVMs.

The patient fulfilled three out of four Curaçao criteria for a definite diagnosis of HHT. HOHF was attributed to chronic volume overload resulting from arteriovenous shunt secondary to large hepatic AVMs, which also led to a severe RH enlargement.

Heart failure medications were initiated with a beta-blocker (metoprolol, 50 mg/d), a sodium/glucose cotransporter-2 inhibitor (empagliflozin, 10 mg/d), mineralocorticoid receptor antagonist (spironolactone, 200 mg/d) and an oral angiotensin receptor-neprilysin inhibitor (sacubitril/valsartan, 48/52 mg/d), which was later stopped due to no evident clinical benefit. Since there was persistent peripheral and central volume overload despite diuretic therapy, peritoneal dialysis was started as a volume overload control strategy with adequate symptom control. Anemia from iron deficiency secondary to HHT was corrected and genetic counseling was offered too. Liver AVM embolization was intended, however given their large size it was not technically feasible. The patient began pre-liver transplantation evaluation. Eight weeks after index hospitalization an episode of refractory cardiogenic shock aggravated by gram negative bacilli bacteremia led to the patient’s death.

## 3. Discussion

Our patient met three out of four Curaçao criteria for HHT, as she presented a history of recurrent epistaxis, mucocutaneous telangiectasias and multiple AVMs (except for a first-degree relative with HHT) [14]. This allowed us to determine her definite diagnosis. Genetic testing is not essential for diagnosis because of the criteria’s high detection rate (up to 97% for ENG variant). For patients with a non-definite diagnosis, the 5 gene HHT panel (assessing for pathogenic mutations in ENG, ACVRL1, MADH4, RASA1, and BMP9) has a clinical sensitivity of approximately 87% [15].

Although hepatic involvement in HHT has been reported in up to 74% of patients, it tends to be symptomatic in less than 10% [15,16]. Hepatic AVMs can manifest as portal hypertension, gastro-esophageal varices, and ascites (in case of connecting hepatic artery and portal vein) and alternatively, HOHF (secondary to connection between hepatic artery and hepatic veins) [13,17].

HOHF, defined as signs and symptoms of systemic or pulmonary venous congestion with an elevated cardiac index on right heart catheterization (usually >4 L/min/m^2^), is an unusual phenotype of HF associated with decreased systemic vascular resistance [12,18]. It remains poorly understood and represents a diagnostic and therapeutic challenge since standard therapies for HF (vasodilators and inotropes) can be detrimental. Potential etiologies include obesity, cirrhosis, arteriovenous shunts (usually related to hemodialysis fistulas or HHT), and less commonly thiamine deficiency, myeloproliferative neoplasms and lung diseases [12]. In AV shunts, the amount of arterial blood passing directly into the venous system must exceed approximately 20% of the cardiac output to produce significant hemodynamic disturbances [13,16,19].

HOHF due to HHT has been associated with worse outcomes, probably because of the lack of information and clinical trial data regarding its treatment. As of today, therapeutic options are intended to treat complications such as volume overload and iron deficiency anemia, a common complication of chronic telangiectasia bleeding. The treatment of HOHF is usually conservative and diuretics are the pillar of management [16,20]. Dietary restriction of salt and water is recommended [20]. Anecdotal cases have been treated with angiotensin receptor blockers or betablockers; however, they have not been systematically evaluated [16]. Intravenous vasoconstrictor adrenergic drugs may be useful in short regimens while treatment of the underlying cause of low systemic vascular resistance is ongoing [20]. For iron deficiency anemia, oral iron is advised as the initial therapeutic consideration. Intravenous supplementation should be considered in patients presenting with severe anemia and in whom oral replacement is expected to be insufficient. RBC transfusions follow the same recommendations as in other patients and in all cases hematology consultation is counseled [21].

AVM local therapy with embolization, banding, or surgical ligation is suggested mainly in patients with large vascular malformations, with partial or complete reversibility of HOHF but significant morbidity and mortality [19]. Liver transplantation can be considered in severe cases as a curative option [19]. Recently, anti-angiogenic therapies (Bevacizumab, PI3 Kinase inhibitors, tyrosine kinase inhibitors) or BMP9/10 signaling pathway inhibitors (Tacrolimus, Sirolimus) have had proven clinical efficacy in treating bleeding and reducing AVM size in small series, with ongoing controlled trials [22,23].

On the other hand, the broad diagnosis of atrial echocardiographic abnormalities and cardiac structural distortion secondary to high output can be misleading at imaging evaluation. Multiple bands and bandlike structures can be found within the cardiac chambers, special among the RA. The knowledge regarding the appearances that these and other band and band-like structures may present is important in differentiating between normal structures (such as the crista terminalis), normal variants (such as a taenia sagitalis or a Chiari network) and aberrant structures and pathologic entities (such as CTD) [24]. Multimodality imaging plays an important role in the evaluation of these structures. Continuous volume overload and PH can lead to severe RH dilation and dysfunction, as was this patient´s case. Atrial RA enlargement can induce Eustachian valve prominence mimicking a dividing membrane as seen in CTD, a rare congenital condition usually associated with other right-side defects that presents with various clinical signs depending on the degree of flow obstruction [25].

## 4. Conclusions

HHT is a rare genetic condition characterized by AVM at multiple locations; cardiovascular manifestations include HOHF secondary to left-to-right blood shunting that can induce diverse functional and structural cardiovascular changes, with scarce evidence regarding therapy, making it a challenging condition to diagnose and treat.

## Data Availability

Not applicable.
